# Articulation of three core metabolic processes in Arabidopsis: Fatty acid biosynthesis, leucine catabolism and starch metabolism

**DOI:** 10.1186/1471-2229-8-76

**Published:** 2008-07-11

**Authors:** Wieslawa I Mentzen, Jianling Peng, Nick Ransom, Basil J Nikolau, Eve Syrkin Wurtele

**Affiliations:** 1 CRS4 Bioinformatics Laboratory, Loc. Piscinamanna, 09010 Pula (CA), Italy; 2 Department of Genetics, Development and Cell Biology, Iowa State University, Ames, IA 50011, USA; 3 Department of Biochemistry, Biophysics and Molecular Biology, Iowa State University, Ames, IA 50011, USA

## Abstract

**Background:**

Elucidating metabolic network structures and functions in multicellular organisms is an emerging goal of functional genomics. We describe the co-expression network of three core metabolic processes in the genetic model plant *Arabidopsis thaliana*: fatty acid biosynthesis, starch metabolism and amino acid (leucine) catabolism.

**Results:**

These co-expression networks form modules populated by genes coding for enzymes that represent the reactions generally considered to define each pathway. However, the modules also incorporate a wider set of genes that encode transporters, cofactor biosynthetic enzymes, precursor-producing enzymes, and regulatory molecules. We tested experimentally the hypothesis that one of the genes tightly co-expressed with starch metabolism module, a putative kinase AtPERK10, will have a role in this process. Indeed, knockout lines of AtPERK10 have an altered starch accumulation. In addition, the co-expression data define a novel hierarchical transcript-level structure associated with catabolism, in which genes performing smaller, more specific tasks appear to be recruited into higher-order modules with a broader catabolic function.

**Conclusion:**

Each of these core metabolic pathways is structured as a module of co-expressed transcripts that co-accumulate over a wide range of environmental and genetic perturbations and developmental stages, and represent an expanded set of macromolecules associated with the common task of supporting the functionality of each metabolic pathway. As experimentally demonstrated, co-expression analysis can provide a rich approach towards understanding gene function.

## Background

Biological systems are characterized by their capacity to achieve net metabolic inter-conversions while maintaining homeostasis in the face of environmental and developmental cues. This capacity is hard-wired into the genetic blueprint of an organism, and is manifested by the controlled expression of the genetic potential of the organism's genome as it responds to divergent signals and prompts. Mechanisms that control the expression of an organism's genetic potential include those that regulate gene transcription, RNA processing, stability and translation, and processing of polypeptides and their assembly into complexes. Some of these complexes are enzyme catalysts, whereas others are structural or regulatory. A *metabolic network *in its broadest sense can be defined as encompassing the collection of catalytic, structural and regulatory genes, which are expressed as mRNAs, proteins and metabolites that work in coordination to achieve net metabolic conversions.

Advances made over the last decade in the area of functional genomics have provided an increasing ability to globally profile genome expression at the level of RNAs, proteins and metabolites. These data define the transcriptome, proteome and metabolome, respectively. It is conceptually possible to identify metabolic networks from experimental data that reveal correlations in abundance of sub-group of molecules (mRNAs, proteins/protein complexes, or metabolites). Given that the behavior of an organism can be regulated by multiple mechanisms that impact the transcriptome, proteome and metabolome, it is significant to ask the extent to which the transcriptome can reveal metabolic networks. In the unicellular eukaryote *Saccharomyces cerevisiae*, which offers the advantage of cell populations that are homogenous and a relatively simple genome with only 6,608 genes (SGD project "Saccharomyces Genome Database", January 13, 2008 [1]), the operation of metabolic networks, including glycolysis and purine metabolism, has been revealed from transcriptome datasets alone [2-4]. But can such metabolic networks be detected in organisms with a larger, more complex genome, or in multicellular organisms, where evidence for metabolic networks can be swamped by noise associated with cellular differentiation?

The pathway-guided approach to examine the correspondence between the known metabolic pathway and the organization of metabolic processes learned from expression data is to select sets of genes for pathways and search for significant co-expression within each pathway. This method has been used to reveal transcriptional co-expression of genes belonging to the same metabolic pathway across different tissues in complex organisms, e.g., Krebs cycle enzymes in frog [5] or lactose biosynthesis enzymes in mouse [6]. Another, module-guided, approach is to classify a set of genes according to their expression patterns, and study the correspondence of these clusters with identifiable biological processes. This approach was used to reveal 44 modules that correspond to several metabolic pathways in *C. elegans*, including lipid and amino acid biosynthesis [7].

In recent years, wealth of expression data emerged and several online repositories for microarray data have been created, including NASCArrays [8], Genevestigator [9], PLEXdb [10], MetaOmGraph [11], ArrayExpress [12], Vanted [13], Virtual Plant [14], ATTED-II [15], Arabidopsis Coexpression Data Mining Tool [16], MapMan [17], and PageMan [18]. The availability of the data querying the transcriptome across the diverse conditions allows for the creation of the compendium of the biological responses to many perturbations, that may guide the interpretation of the microarray experiments and the annotation of the new genes [19-22]. Because it has become technically possible to profile the entire transcriptome of an organism (it is still a technical challenge to determine the proteome and metabolome), we tested the feasibility of using only transcriptomics data to reveal the operation of metabolic networks in Arabidopsis. A study by Gachon et al. has shown that genes for enzymes of indole, phenylpropanoid and flavonoid biosynthesis in Arabidopsis are co-expressed [23]. Similar co-expression has been shown for enzymes for synthesis of cellulose and other cell wall components [24-26] and in isoprenoid biosynthesis pathways [27]. An analysis of the transcription coordination of 1,330 Arabidopsis genes implicated in metabolic pathways in AraCyc revealed that the co-expression among genes from the same pathway is higher, than among genes from different pathways [28]. Several metabolic processes could be also identified as coherent subnetworks in the recent analysis of Arabidopsis gene network, based on the compendium of the expression profiles [29].

Here, we focus on three core metabolic pathways of Arabidopsis: fatty acid biosynthesis, starch metabolism and leucine catabolism, and analyze their expression in the context of the 22,746 genes represented on the Affymetrix ATH1 chip. Our approach unites the pathway-guided and module-guided methods in that we group the pre-selected set of known and putative genes from pathways into modules, and subsequently assign the function to those putative candidates that are within the module. We exploit the assumption that a node in a network can be hypothesized to have a similar attribute (e.g., function, or location, if nodes represent genes) as the majority of the nodes in its neighborhood of some radius. This assumption has recently gained popularity as the way of assigning function to otherwise unknown genes [28,30,31]. The co-expression network created from among the genes from these pathways forms modules that include enzymes for reactions within each pathway, suggest extensions of catalytic steps in the pathway, and also allow for identification of transporters, cofactors and regulatory molecules associated with the module. Indeed, one of the genes identified in the starch module had been shown independently [32] to be involved in regulation of starch metabolism. We used knock out mutants to experimentally evaluate a second putative regulatory gene in the starch module.

## Results and discussion

### Fatty Acid Biosynthesis, Starch Metabolism and Leucine Catabolism form Comprehensive Modules

**Figure 1:**
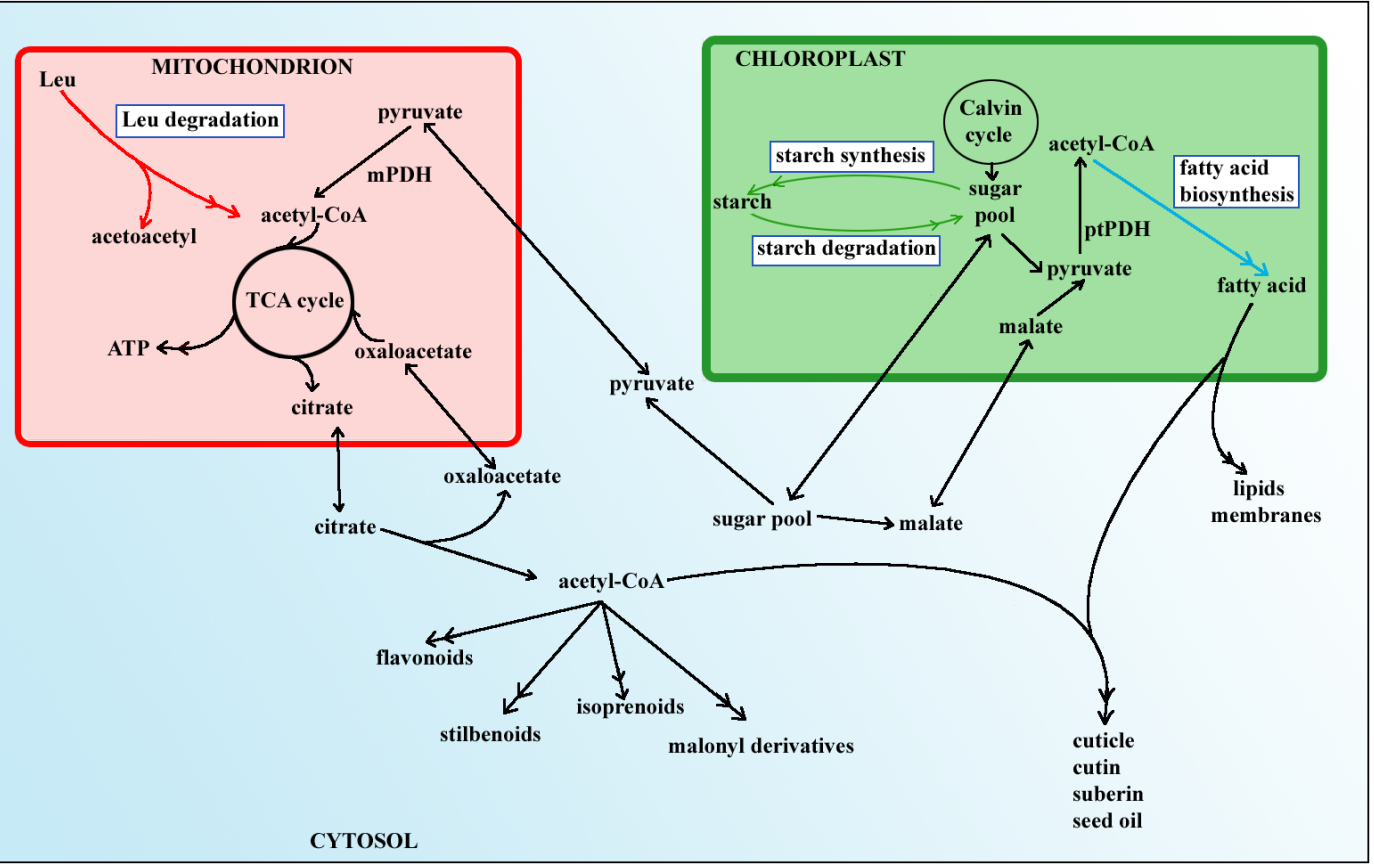
Metabolic context of Arabidopsis leucine catabolism, starch metabolism and fatty acid biosynthesis pathways (blue rectangles).

In order to determine how co-expression networks extend across metabolic processes, we selected three core metabolic pathways for analysis: fatty acid biosynthesis, starch metabolism, and mitochondrial leucine catabolism (Fig. 1). These pathways were selected in part because of the authors' extensive knowledge of their structure and biological implications (e.g., [33-39]). Fatty acid biosynthesis is the process by which acetyl-CoA is used to generate acyl moieties that are used as components of cellular membranes, signaling molecules, and molecules that store energy (e.g., seed oils) [40]. Starch is the principal polymeric storage form of glucose, and is stored either transiently in leaves or long-term in seeds [41]. Leucine catabolism provides an alternate source of acetyl-CoA to sustain respiration and metabolic processes in the absence of photosynthesis [42]. We assembled a list of genes with a demonstrated or putative function in these three metabolic pathways; these genes encode not only the enzymes of the "textbook pathway", but also enzymes important for synthesis of cofactors and transporters that are thought to be involved in these processes. Specifically, a set of 126 genes associated with these pathways was identified based upon one of three criteria: 1) genes that were demonstrated by experimental evidence to belong to one of these pathways; 2) genes assigned "putative" functionality in one of these pathways, based on sequence similarity and subcellular localization; or 3) genes encoding transporters and enzymes synthesizing co-factors required for these pathways [see Additional file 1].

**Figure 2:**
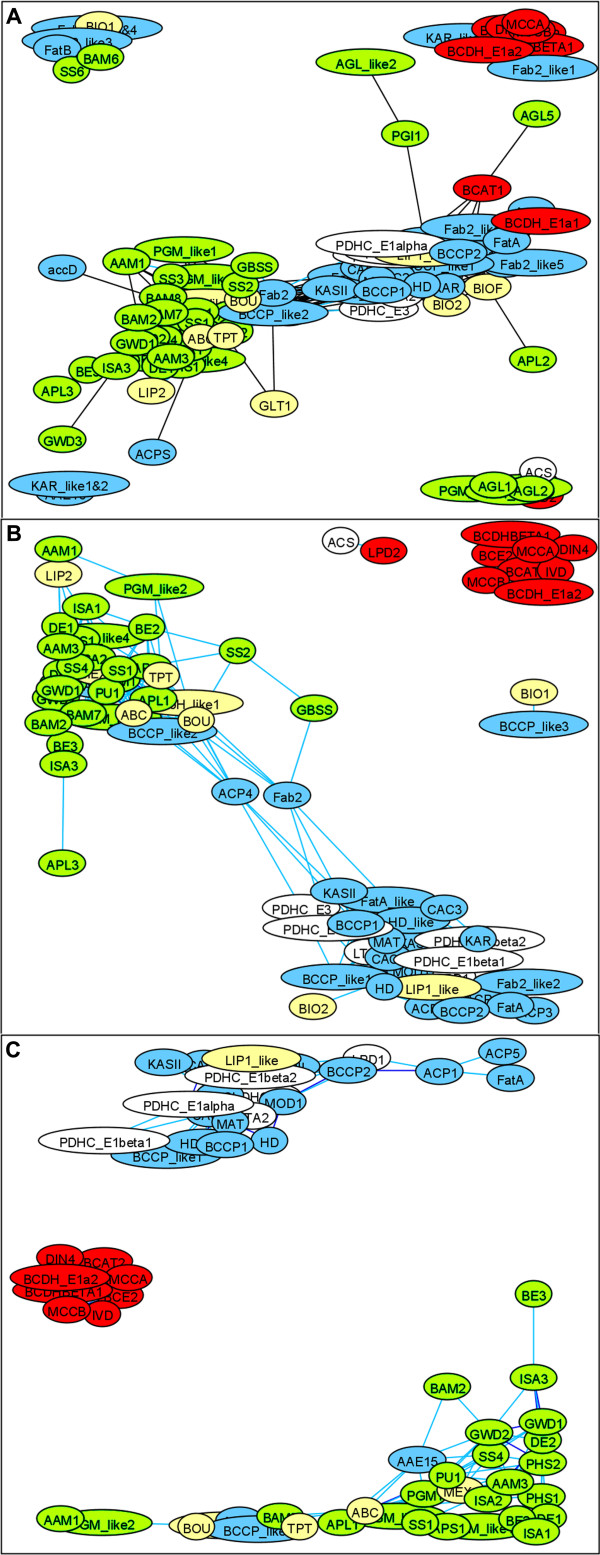
**Coexpression of genes within three core metabolic pathways**. The entities with correlations above the threshold are connected with an edge; networks at three thresholds of Pearson correlation are compared: **A**, 0.5; **B**, 0.6; and **C**, 0.7. With increasing correlation threshold, the within-pathway links emerge from noisy inter-pathway connections. Node colors represent the metabolic function assigned to each gene (blue: fatty acid synthesis, green: starch metabolism, red: leucine catabolism, yellow: transport or cofactor synthesis, white: acetyl-CoA generation) [see Additional file 1 for gene names]. The networks layouts were produced by GraphExplore software. The most densely crowded nodes indicate genes with the highest co-expression. Within each of these three co-expression networks the number of links between genes from the same metabolic pathway is significantly larger than in randomly generated networks with similar link structure. Isolated nodes not shown.

**Figure 3:**
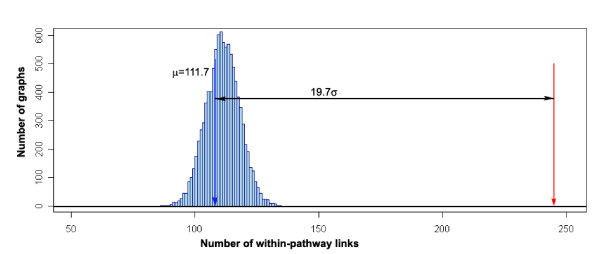
**Enrichment of edges joining genes from the same pathway in the graph in Fig. 2B (red arrow), as compared to random graphs (blue histogram)**. Histogram shows distribution of numbers of links joining genes from the same pathway in each of 10,000 random graphs with the same links structure as the original graph (μ = 111.7; σ = 6.8). Red arrow denotes number of within-pathway links (245) based on expression data (from Fig. 2B graph), blue arrow denotes mean number of within-pathway links (111.7) in randomly obtained graphs. Total number of links in each graph is 444.

Publicly available Affymetrix ATH1 chip-transcriptomic datasets comprising of 956 biological samples were used to infer patterns of transcript co-accumulation for the 22,746 Arabidopsis genes that are represented on this chip (see Methods section for details). These data are drawn from 72 experiments from NASCArrays [43] and PLEXdb [44] that represent a wide range of developmental, and environmental and genetic perturbations on Arabidopsis [see Additional file 2]. Data with poor reproducibility were discarded, and the data was normalized to the same mean and range (available at MetaOmGraph [11]). We calculated the values of Pearson pair-wise correlation across these data for the set of 126 selected genes. The results were visualized as a graph, such that genes form the nodes, which are joined by an edge if the value of correlation between them is higher than selected threshold. Using Pearson correlation thresholds of 0.5, 0.6 and 0.7 yields three co-expression networks of 107, 77 and 62 genes, connected by 733, 444, and 204 edges, respectively (Fig. 2). The positioning of the nodes (genes) in these networks was produced by graph-layout software that places highly interconnected nodes close together [45]. Thus, the absolute position of the node on the plot has no meaning, only its relative position versus other nodes: the most closely crowded nodes indicate genes with the highest co-expression. Within each of these co-expression networks the number of correlations between genes from the same metabolic pathway is significantly larger than in randomly generated networks with similar link structure (specifically, in the network at threshold 0.6, the proportion of within-pathway edges is 245/444, versus a mean of 112/444 in randomly rewired networks; this is equal to a difference of ~20 standard deviations; Fig. 3). As the correlation threshold is increased from 0.5 to 0.7, three distinct closely-interconnected clusters emerge, whose member genes closely correspond with the three metabolic pathways that were the focus of this study. We refer to the three closely-interconnected clusters identifiable in Fig. 2B as modules.

Starch is a complex molecule, whose metabolism comprises a network with many genes of known catalytic function but unclear physiological function [46,47]. Indeed, several genes may function both in the catabolism and biosynthesis of starch (e.g., pullulanase [48]). At the 0.6 correlation level, the starch metabolism module contains 28 genes encoding all the known enzymes of starch metabolism. The module incorporates genes encoding proteins, for which, to date, there is no experimental evidence of involvement in starch metabolism, for example beta-amylase At2g32290 (BAM2), and three phosphoglucomutase-like proteins (At4g11570, PGM-like2; At1g70730, PGM-like3 and At1g70820, PGM-like4). On the other hand, alpha-glucosidase, an enzyme which historically had been considered to be involved in starch degradation, although recent experimental data from potato indicate otherwise [49], is not correlated with the Arabidopsis starch metabolic module (Fig. 2B). Although the processes of starch synthesis and degradation are temporarily separated over diurnal cycles in Arabidopsis leaves (reviewed in [50]; [51]), as is the transcription of many of the starch metabolic genes ([52,53]; Foster and Wurtele, unpublished data), this is not reflected in the co-expression network of Arabidopsis; rather the two processes cluster together in a single module. Two factors in particular may contribute to starch synthesis and degradation transcripts being in a common module. First, Arabidopsis doesn't have a major starch-storage organ such as a tuber, thus an organ of Arabidopsis that is highly capable of starch synthesis may also be capable of a rapid shift to starch degradation, and the overall levels of all the relevant transcripts may be high. Indeed, this is the case for the diurnal fluctuations of the transcripts. Second, as several of the starch metabolic enzymes appear to function in both starch degradation and synthesis [48] (indeed the full extent to which this is true is not yet known), expression of such genes may be associated with both starch degradation and starch synthesis.

**Figure 4:**
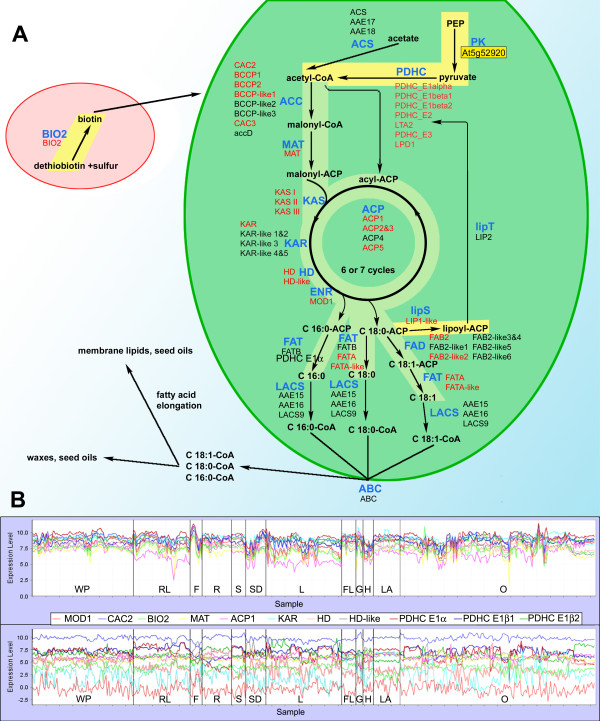
**Coexpression of the genes for fatty acid biosynthesis**. **A **– Fatty acid biosynthesis in chloroplast. Candidate genes for enzymatic function (enzyme names in blue) are shown in black or red font; genes whose Locus ID is in red are co-expressed. These genes form connected network in Fig. 2B and encode all enzymes required for the biosynthesis of 18-carbon fatty acids from acetyl-CoA (sequence of reactions on the lighter green background), as well as genes for producing of acetyl-CoA substrate, and for biosynthesis of biotin and lipoic acid, obligate cofactors for the acetyl-CoA carboxylase and for pyruvate dehydrogenase (sequence of reactions on the lighter yellow background). Gene for pyruvate kinase (on yellow background, identified by search for genes co-expressed with MOD1), required for production of pyruvate, is also co-expressed with genes from the fatty acid biosynthesis pathway [see Additional file 1 for the correspondence between gene names and Locus IDs]. **B **– Expression profiles of the 11 most tightly co-expressed genes from fatty acid biosynthesis module across 956 microarray chips (top panel) and, for comparison, expression profiles of the 11 randomly chosen genes (bottom panel). Experiments with tissues and organ samples are denoted: WP, whole plant; RL, rosette leaf; F, fruit; R, root; S, shoot; SD, seed; L, leaf; FL, flower; G, male gametophyte; H, hypocotyls; LA, leaf apex; O, other. **ABC**, acyl transporter; **ACC**, heteromeric acetyl-CoA carboxylase; **ACS**, acetyl-CoA synthetase; **BIO2**, biotin synthase; **ENR**, enoyl-[ACP] reductase; **FAD**, fatty acyl-[ACP] desaturase; **FAT**, fatty acyl-[ACP] thioesterase; **HD**, 3-hydroxyacyl-[ACP] dehydratase; **KAR**, 3-ketoacyl – [ACP] reductase; **KAS**, 3-ketoacyl-[ACP] synthase; **LACS**, plastidial long-chain acyl-CoA synthetase; **lipS**, lipoate synthase; **lipT**, lipoate transferase; **MAT**, malonyl-CoA:ACP transacylase; **PDHC**, pyruvate dehydrogenase complex; **PK**, pyruvate kinase.

The fatty acid synthesis module contains genes encoding the complete set of enzymes required for the biosynthesis of 18-carbon fatty acids from acetyl-CoA: three nuclear-encoded subunits of acetyl-CoA carboxylase (CAC2, CAC3, BCCP1, BCCP2, and BCCP1-like), malonyl-CoA:ACP transacylase (MAT), acyl carrier protein (ACP1, ACP2&3 and ACP5), beta-ketoacyl-[ACP] synthases (KAS I, KAS II and KAS III), beta-ketoacyl-[ACP] reductase (KAR), beta-hydroxyacyl-[ACP] dehydratase (HD and HD-like), enoyl-ACP reductase (MOD1), stearoyl-[ACP] desaturase (FAB2 and FAB2-like2) and acyl-[ACP] thioesterases (FATA and FATA-like) (Fig. 4A). This module also contains all genes for the four subunits of plastidic pyruvate dehydrogenase, which produces acetyl-CoA (the precursor for fatty acid biosynthesis) from pyruvate. The apparent co-expression of the pyruvate dehydrogenase genes with fatty acid biosynthesis may indicate the direction of the major metabolic flux of acetyl-CoA, even though there are other fates of plastidic acetyl-CoA (e.g., biosynthesis of glucosinolates and amino acids, such as leucine and cysteine). Interestingly, the module contains genes for the final reaction in biotin biosynthesis [54-56] and for lipoic acid biosynthesis, obligate cofactors of acetyl-CoA carboxylase and pyruvate dehydrogenase, respectively. In contrast, the acetyl-CoA synthetase (ACS) genes do not show any expression correlation, consistent with experimental evidence that this enzyme is not required to provide acetyl-CoA for fatty acid biosynthesis [33,57,58].

**Figure 5:**
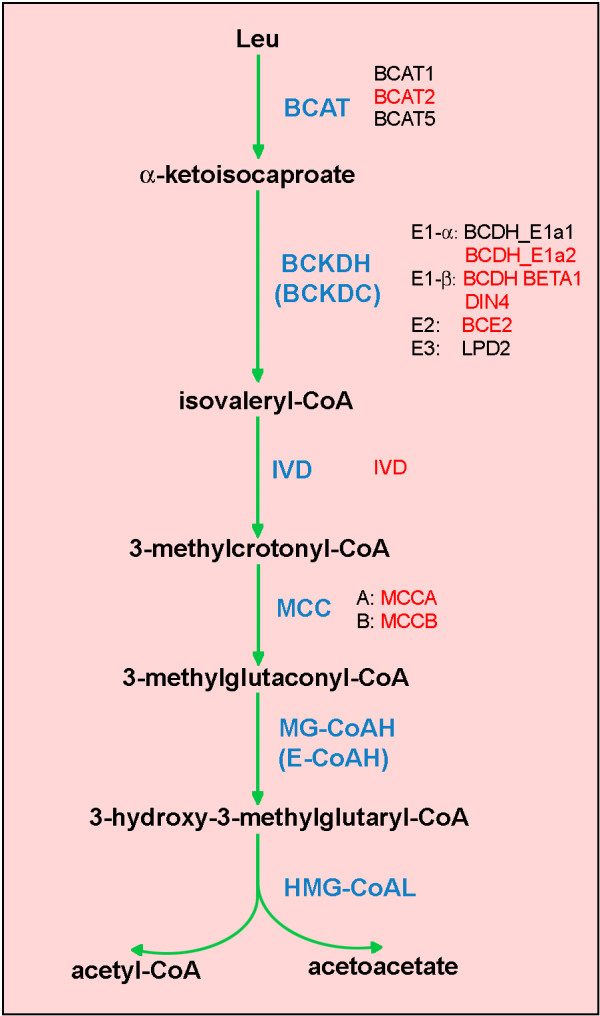
**Leucine degradation pathway in mitochondria**. The enzyme names are in blue, gene names in black/red [see Additional file 1] [42,84]. Genes that are co-expressed are depicted in red font. **BCAT**, branched-chain amino acid aminotransferase; **BCKDH (BCKDC)**, branched-chain alpha-keto acid dehydrogenase; **IVD**, isovaleryl-CoA dehydrogenase; **MCC**, methylcrotonyl-CoA carboxylase; **MG-CoAH (E-CoAH)**, methylglutaconyl-CoA hydratase (enoyl-CoA hydratase); **HMG-CoAL**, hydroxymethylglutaryl-CoA lyase. The candidate genes for enzymes catalyzing two terminal reactions have not been identified.

The leucine degradation module contains genes for the four known enzymes of mitochondrial leucine catabolism (Fig. 5): branched-chain amino acid aminotransferase (BCAT), three out of four subunits of branched-chain alpha-keto acid dehydrogenase (BCDH), isovaleryl-CoA dehydrogenase (IVD) and methylcrotonyl-CoA carboxylase (MCC). Only a single BCAT is included in the module (BCAT2, At1g10070). This gene is not the one postulated by Schuster and Binder [59] to participate in leucine degradation. Consistent with these results, seven out of the eight genes in our module (except IVD) form a coherent subnetwork in the Arabidopsis gene network based on partial correlation [29].

### Modules are Identifiable in the Space of All Gene Transcripts

To test whether these pathway-specific modules can be detected in the context of the expression of the entire genome, and to determine whether genes other than those encoding catalytic functions of the pathway are co-regulated, we calculated Pearson correlations between individual pathway-specific hub genes and the 22,746 genes represented on the Affymetrix chip. The genes used as hubs for each metabolic pathway are: At2g05990, encoding the enoyl-ACP reductase that is uniquely required in *de novo *fatty acid biosynthesis [60]; At2g40840, starch metabolism hub gene coding for disproportionating enzyme [61]; and the leucine catabolism hub gene, At4g34030, which encodes the β subunit of methylcrotonyl-CoA carboxylase [39,42] (Table 1).

**Table 1:** Genes from 22 k Arabidopsis ATH1 array that have the highest correlation across 965 chips with the hub genes.

Hub Gene: MCCB, At4g34030, methylcrotonyl-CoA carboxylase, subunit B, **leucine catabolism**		
Corr.	GeneID	Description

0.9	**At1g03090**	**MCCA, methylcrotonyl-CoA carboxylase, subunit A**
0.84	**At3g06850**	**BCE2, E2 subunit of branched chain alpha-ketoacid dehydrogenase**
0.84	**At3g13450**	**DIN4, E1 beta subunit of branched-chain alpha-keto acid dehydrogenase**
0.82	At2g43400	electron transfer flavoprotein: ubiquinone oxidoreductase family protein
0.8	**At3g45300**	**IVD, isovaleryl-CoA dehydrogenase**
0.8	At4g35770	Senescence-associated protein (SEN1)
0.79	At5g21170	AMP-activated protein kinase
0.78	At1g08630	threonine aldolase
0.78	At1g15040	glutamine amidotransferase-related
0.77	**At1g10070**	**BCAT2, branched-chain amino acid transaminase**
0.74	At4g28040	nodulin MtN21 family protein
0.73	At3g47340	glutamine-dependent asparagine synthetase 1 (ASN1)
0.73	**At1g21400**	**E1 alpha subunit of branched-chain alpha-keto acid dehydrogenase, BCDH_E1a2**
0.72	At1g18270	ketose-bisphosphate aldolase class-II family protein
0.71	At5g49450*	bZIP family transcription factor
0.71	**At1g55510**	**BCDH BETA1, E1 beta subunit of branched-chain alpha-keto acid dehydrogenase**
0.7	At3g49790	expressed protein

Hub Gene: DE2, At2g40840, disproportionating enzyme, **starch metabolism**		

Corr.	GeneID	Description

0.89	**At3g46970**	**PHS2, starch phosphorylase**
0.85	**At1g10760**	**GWD1, glucan water dikinase**
0.85	At3g52180*	SEX4, protein tyrosine phosphatase/kinase
0.8	**At1g03310**	**ISA2, isoamylase DB**
0.79	**At4g09020**	**ISA3, isoamylase DB**
0.76	At1g06460	31.2 kDa small heat shock family protein/hsp20 family protein
0.74	**At5g26570**	**GWD2, glucan water dikinase**
0.74	**At3g29320**	**PHS1, starch phosphorylase**
0.73	At5g59130	subtilase family protein
0.73	At2g39920	acid phosphatase class B family protein
0.72	**At1g69830**	**AAM3, alpha-amylase**
0.72	At1g67660	expressed protein
0.71	At3g18500	similar to endonuclease/exonuclease/phosphatase family protein
0.7	At2g31040	ATP synthase protein I-related
0.7	At4g33490	nucellin-like protein

Hub Gene: MOD1, At2g05990, enoyl reductase, **fatty acid biosynthesis**		

Corr.	GeneID	Description

0.86	**At1g34430**	**PDHC_E2, acetyltransferase subunit of pyruvate dehydrogenase**
0.86	**At5g10160**	**HD, 3-hydroxyacyl-[ACP] dehydratase**
0.84	**At5g35360**	**CAC2, biotin carboxylase subunit of heteromeric acetyl-CoA carboxylase**
0.83	**At5g46290**	**KASI, 3-ketoacyl-[ACP] synthase I**
0.8	**At2g30200**	**MAT, malonyl-CoA:ACP transacylase**
0.8	**At3g16950**	**LPD1, lipoamide dehydrogenase subunit of pyruvate dehydrogenase**
0.79	**At1g62640**	**KAS III, 3-ketoacyl-[ACP] synthase III**
0.78	At1g53520^$^	chalcone-flavanone isomerase-related
0.77	**At3g25860**	**LTA2, acetyltransferase subunit of pyruvate dehydrogenase**
0.77	At5g52920	pyruvate kinase
0.76	**At5g16390**	**BCCP1, biotin carboxyl carrier subunit of heteromeric acetyl-CoA carboxylase**
0.75	**At5g08415**	**LIP1-like, lipoate synthase**
0.73	**At2g22230**	**HD-like, 3-hydroxyacyl-[ACP] dehydratase**
0.72	**At1g30120**	**PDHC_E1beta2, E1 beta subunit of plastidic pyruvate dehydrogenase**
0.72	**At5g15530**	**BCCP2, biotin carboxyl carrier subunit of heteromeric acetyl-CoA carboxylase**
0.71	**At1g01090**	**PDHC_E1alpha, E1 alpha subunit of plastidic pyruvate dehydrogenase**
0.71	At5g50390*	pentatricopeptide (PPR) repeat-containing protein
0.7	At4g12700	expressed protein

Genes used as the hub (at the top of the table) are methylcrotonyl-CoA carboxylase (MCCB), active in leucine degradation in mitochondria; disproportionating enzyme (DE2), from starch metabolism; and enoyl reductase (MOD1), an enzyme from plastidic fatty acid biosynthesis pathway. Genes that code for proteins predicted to be in the same pathway as the hub gene are in bold font. Potential regulatory genes are marked with an asterisk. Pearson coefficients (> 0.7) were calculated by MetaOmGraph. P-values for enrichment of genes from same pathway as hub are 4.35e-36, 2.02e-15, and 3.1e-23, respectively.

^$ ^This gene product, although annotated as chalcone isomerase-like, appears to have a direct role in binding and possibly transporting fatty acids (Joe Noel and Florence Pojer, personal communication).

Despite the complexities associated with a genome of about 32,000 genes (TAIR7 Genome Release, April 23, 2007 [62]), and the challenge associated with the fact that the RNAs used for the microarray analyses came from plant samples that mix metabolically distinct cellular and tissue compartments, it was still possible to find expression correlations among genes of common metabolic pathways. Specifically, the majority of the genes that show the highest correlation with the fatty acid biosynthesis hub gene, the starch metabolism hub-gene, and the leucine catabolism hub-gene are the structural genes that code for the enzymes required in each of these metabolic pathways (Table 1). Similarly, for every gene in the module, we found genes correlated with it (above the set threshold) among 22,746 genes on ATH1 array and checked how many of those correlated genes are also in the same module ("in" links) and how many are not in module ("out" links). We found that at 0.7 threshold, for 19 genes out of 22 in the fatty acid biosynthesis module, the number of "in" links is higher than the number of "out" links, and the same is true for 7 out of 8 leucine catabolism genes, and 9 out of 16 starch metabolism genes at the threshold of 0.8. Thus, these genes are preferentially interconnected among themselves with fewer connections to other genes, which defines them as a module in the context of the Arabidopsis genome.

Analysis of co-expression also provides means of querying the consequence of genome duplications. As with other plant genomes, Arabidopsis has undergone whole-genome duplications (polyploidization), followed by radical gene silencing, gene turnover, and expansion of gene families, the most recent of which is thought to have occurred about 25 million years ago [63,64]. In our analysis, At5g52920, one of the fourteen Arabidopsis genes that code for pyruvate kinase, is highly correlated with the fatty acid biosynthesis hub-gene (Table 1). Pyruvate kinase catalyzes the conversion of phosphoenolpyruvate to pyruvate, the immediate precursor of the acetyl-CoA used for fatty acid biosynthesis [65]. These data, therefore, help to constrain the complexity associated with the polyploidization, and indicate that of the fourteen pyruvate kinase genes that occur in the genome, At5g52920 is most likely the one predominantly utilized for fatty acid biosynthesis.

In addition, the co-expression of pyruvate dehydrogenase and pyruvate kinase with the fatty acid biosynthesis genes implies that the functional pathway commonly thought of as "fatty acid synthesis" (acetyl-CoA to fatty acids) could be expanded to begin with phosphoenolopyruvate (PEP), the substrate for pyruvate kinase (Fig. 4A).

### Potential Regulatory Genes Identified as Coexpressing with Metabolic Pathways

A set of potential regulatory genes whose expression patterns highly correlate with each of the pathway-specific hub-gene was identified in this analysis (genes marked with an asterisk, Table 1). Although these correlations cannot be extrapolated to imply a causative role, identification of candidate regulatory genes delineates hypotheses and suggests experimental approaches for identifying the regulatory function of these genes.

**Figure 6:**
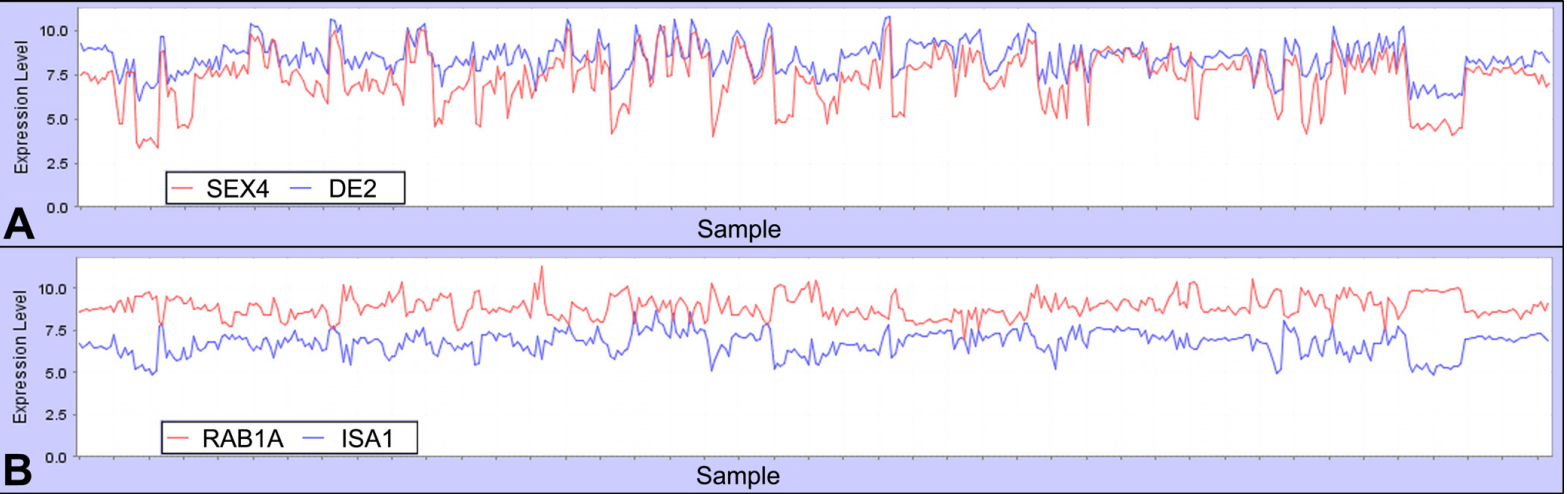
**Correlation of regulatory genes with starch metabolism module in 956 microarray chips**. **A **– Positive correlation. SEX4, protein tyrosine phosphatase/kinase; DE2, disproportionating enzyme. Correlation = 0.85. **B **– Negative correlation. RAB1A, a RAB-like GTPase; ISA1, isoamylase. Correlation = -0.54.

One such putative regulatory gene encodes a protein tyrosine phosphatase/kinase gene SEX4 (At3g52180) (Table 1 and Fig. 6A). Its presence in this module would lead us to hypothesize an involvement in regulation of starch metabolism. Indeed, this gene was recently shown experimentally to have a regulatory role in starch metabolism; knockout alleles of At3g52180 have a starch excess phenotype [32]. Similarly, a gene encoding a putative protein kinase (At1g26150, AtPERK10 [66]) is correlated with genes in the starch metabolism module. We directly checked whether this gene might play a role in regulation of starch synthesis.

**Figure 7:**
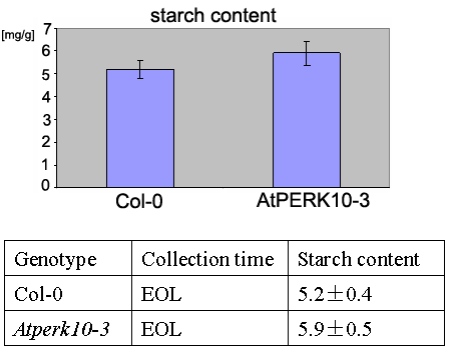
**Starch content of AtPERK10 knockout plants**. Starch content of the wild type phenotypes and the knockouts of the AtPERK10 gene (At1g26150), a putative protein kinase whose expression correlates with the expression of starch metabolism genes. The mutant plants have 13% more starch than WT plant (p-value = 0.045). *Atperk10-3 *is in Col-0 background. Col-0 is a wild type sibling. EOL: samples taken at the end of the light phase.

We obtained three independent alleles, named *Atperk10-*1, *Atperk10-2*, and *Atperk10-3*, corresponding to T-DNA insertions at gene AtPERK10 locus. Homozygous mutant lines stained for starch using Lugol solution showed an increase in starch accumulation (data not shown). Figure 7 shows a quantitative comparison of starch content in wild type and *Atperk10-3 *homozygous allele.

Interestingly, the starch hub-gene shows a high negative correlation with two Rab-like, GTPase-coding genes (At5g47200; RAB1A and At4g17530; RAB1C; Fig. 6B). In eukaryotes, proteins from this family of small GTP-ases are regulatory and act as signaling molecules in diverse processes [67]. The Arabidopsis protein that is phylogenetically the closest homolog to Rab-like GTPase is RAB1B (At1g02130), which is localized to the ER, and as in mammalian systems, it is important for Golgi apparatus development [68]. RAB1A and RAB1C are predicted to be secretory and might have function similar to that of RAB1B, or some novel plant-specific regulatory function, which appears to be negatively correlated with starch metabolism.

The fatty acid synthesis module contains a pentatricopeptide repeat-containing protein (encoded by At5g50390). Because such proteins are thought to participate in modulating the stability of organelle transcripts [69], this correlation may indicate a role for this gene in coordinating nuclear-plastidic interactions for regulating fatty acid biosynthesis, via, for example, the control of the plastid-encoded subunit gene of acetyl-CoA carboxylase (*accD*, AtCg00500). This is the plastid-encoded biochemical function that is most directly involved in fatty acid biosynthesis.

The leucine catabolism module contains a putative transcriptional regulator, At5g49450, which may code for a bZIP family transcription factor.

### Hierarchical Modularity of Catabolism

**Figure 8:**
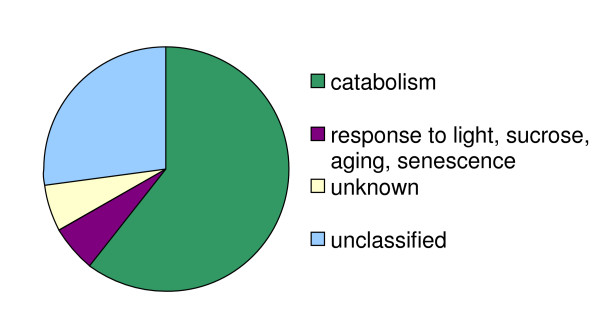
**Higher order catabolic module is revealed from the functional categories of the genes correlated with the leucine catabolism module (Pearson coefficient > 0.5)**. Genes in the module were identified by intersection of lists of all genes that are correlated above 0.5 threshold with each of eight genes from leucine catabolism module.

These transcriptome level analyses provide insights into the hierarchical modularity of Arabidopsis metabolism. Hierarchical organization can be considered the nesting of one, tightly connected module within a larger one, still distinct and separable from the surrounding network. Hierarchical organization is particularly evident with leucine catabolism, which forms a functionally coherent and tightly connected module, nested within a supermodule [see Fig. 8 and Additional file 3].

Hierarchical organization of modules has been observed for metabolic and transcriptional networks of *E. coli *[70-72] and metabolic networks of 42 other organisms [73,74]. Hierarchical modular organization has been postulated as the topology explaining the observed features of biological networks, like scale-free character, high clustering coefficient and short path lengths [74,75]. Such architecture may enable better adaptation of the metabolic fluxes to the changing conditions [74].

For Arabidopsis, the incorporation of leucine catabolism into an overarching catabolic module is observed both on the topological and functional level. From the topological point of view, genes participating in the leucine catabolism form a fully connected subnetwork, while they also exhibit weaker connection to the common larger set of genes. Thus, a smaller, more strongly connected module is nested within the bigger and less interconnected one. From the functional point of view, one function (leucine catabolism) forms a separable part of the larger, more encompassing biological process. The supermodule contains genes that appear to have a broader but common biological task of maintaining cellular energy balance via catabolism. Specifically, the 26 genes most highly correlated with the eight genes in the leucine catabolic module (defined in Fig. 2B) include genes that might be involved in protein turnover (At1g76410), amino acid catabolism (At1g06570, At1g08630, At2g14170, At3g47340, At5g63620, in addition to eight genes from leucine catabolism pathway), carbohydrate catabolism (At1g18270, At5g20250, At5g49360), lipid breakdown (At3g51840, At5g41080) and cell wall degradation (At4g30270). Five genes are annotated as associated with aging, senescence, or response to light or sucrose stimuli (At2g43400, At3g47340, At4g30270, At4g35770, At5g20250). Because plants are photoautotrophs, such catabolic processes may be expected to become critical when photosynthesis cannot maintain cellular energy balance, for example during senescence, seed germination, carbon deprivation or autophagy [37,39,42,76]. Consistent with this concept, we find that the genes within this supermodule are co-induced to highest levels of expression under experimental conditions that are expected to limit photosynthetic carbon fixation and induce catabolic processes for maintaining energy balance (e.g., oxidative, cold, heat and drought stress – experiments from NASCArrays: NASCARRAYS-28, NASCARRAYS-79, NASCARRAYS-70; disruption of plastome-nucleome communication, NASCARRAYS-89; mutations and illumination conditions that affect the phytochrome response, NASCARRAYS-89, NASCARRAYS-124; sucrose starvation, Contento et al. [76] and NASCARRAYS-14; and hexose signaling, NASCARRAYS-40 [8]).

This catabolic supermodule also contains genes with well-defined or partially defined molecular functions, but undefined physiological functionality (e.g. At1g76410, zinc finger protein; At5g21170, protein kinase; At5g16340, AMP-binding protein). The inclusion of these genes within this catabolic supermodule may indicate their broader physiological function in catabolic processes; a hypothesis that can be experimentally tested by new genetic and biochemical analyses.

## Conclusion

This study reveals that transcriptomics data can divulge hitherto unsuspected aspects of metabolic networks in a complex eukaryotic organism. Each of these core metabolic pathways that were targeted for analysis is structured as a co-expressed module whose transcripts co-accumulate over a wide range of environmental and genetic perturbations and developmental stages. These analyses further indicate that modules can be recruited into hierarchical organization (supermodules), hinting at the possibility that supermodules are regulated by common signals that coordinate net metabolic changes within highly interactive networks. The fact that such hierarchical organization is detected from the transcriptome data implies that at least a subset of the higher-order metabolic network may be regulated at the transcript level.

## Methods

### Transcriptomic Data

The experimental and meta-data was obtained from online microarray depositories: NASCArrays [43] and PLEXdb [44]. The data obtained from NASCArrays, from 72 experiments comprising of 956 Affymetrix ATH1 microarray slides, had already been normalized with the Affymetrix MAS 5.0 algorithm to the common mean value of 100. We scaled the data from PLEXdb to set the mean to 100.

The reproducibility of experiments was qualitatively assessed on scatter plots by visual inspection and chips with poor biological replicates were discarded. The remaining biological replicates were averaged to yield 424 samples. Determination of biological replicates (replicates of the same treatment within the experiments) was done manually, based on the slide name and experiment information. Most slides have descriptive names that summarize treatment and the replicate status. Thus, slides that are biological replicates of the same treatment have the same slide name, differing only in "Rep**x**" suffix, e.g.,

Shirras_1–3_Non-Calcicole_Rep1

Shirras_1–4_Non-Calcicole_Rep2

The data was subsequently normalized to the same range by a median absolute deviation (MAD)-based scale normalization method described by Yang et al., [77]. Expression values *x*_*ij *_on microarray chip *j *were multiplied by the factor $\frac{C}{MAD_{j}}$, where *MAD *is defined by

*MAD*_*j *_= median_*i*_{|*x*_*ij*_-median_*i*_(*x*_*ij*_)|}

and the constant C is an arithmetic mean of *MAD*

$$C=\frac{\sum_{j=1}^{n}MAD_{j}}{n}$$

The normalized data is available online [11]. ATH1 probe set-to-Locus ID mapping was obtained from TAIR, March 31^st ^2007 [78,79]. The experimental information is included in Additional file 2.

### Pathway Data

The choice of genes involved in each pathway was guided by: Arabidopsis Lipid Gene Database for fatty acid synthesis genes [80,81]; Arabidopsis Starch Metabolism Network project [82] for starch metabolism; and original references for leucine catabolism [42,83,84]. We further considered each candidate gene based on original references.

### Network of Coexpressed Genes

The 126 genes used in the initial network construction (Fig. 2), including candidates for functions in fatty acid synthesis, starch metabolism and leucine catabolism, are shown in Additional file 1. A correlation matrix was calculated for these genes, using Pearson Product-Moment Coefficient, defined as

$$r_{x,y}=\frac{\sum_{i=1}^{n}(x_{i}-\bar{x})(y_{i}-\bar{y})}{(n-1)\delta_{x}\delta_{y}}$$

The values of Pearson correlations above 0.3 are statistically highly significant (p-value < 0.00001 after Bonferroni correction for multiple testing [85]). We additionally assessed the validity of correlations higher than 0.6 by performing 10,000 permutations of the corresponding data vectors. Correlations that had a permutation-based p-value < 0.0001 (in our case, all pairs of genes examined) were considered for further analysis [see Additional file 4]. The networks in Fig. 2 were constructed by placing an edge between any pair of genes correlated above the threshold. Modules in the network were defined by the grouping produced by the network visualization software.

### Enrichment of Within-Pathway Edges in the Coexpression Network

To test whether there are more correlations between the genes from the same metabolic pathway (i.e. from fatty acid biosynthesis, starch metabolism, or leucine catabolism category) than could be expected by chance, we compared the co-expression network from experimental data (Fig. 2B) to 10,000 computationally generated random networks. In these random networks the total number of the edges and the number of neighbors for each node was set to be the same as in the original graph. A function for generating random graphs was implemented in R. It constructed graphs by random filling of the adjacency matrix, where nodes' degrees were constrained and self-loops not allowed. Indeed, the proportion of within-pathway edges is significantly higher in our coexpression network compared to such proportions in random graphs (245/444 versus a mean of 112/444, which is equal to the difference of ~20 standard deviations, Fig. 3).

### Correlation with Hub Genes

The calculation of Pearson correlations between the three hub-genes (At2g40840, At4g34030, At2g05990) and all other genes on the chip (Table 1) was conducted in our publicly available software, MetaOmGraph [11]. MetaOmGraph is able to handle large datasets with an efficient use of memory and is integrated with other publicly available bioinformatics tools in MetNet Exchange suit [86,87].

The genes belonging to catabolic supermodule [see Fig. 8 and Additional file 3] were identified by intersection of lists of all genes that are correlated above 0.5 threshold with each of eight genes from leucine catabolism module.

### Plant Material

Three independent alleles named *Atperk10-1*, *Atperk10-2*, and *Atperk10-3 *corresponding to T-DNA insertions at gene AtPERK10 locus were found in the mutant collections generated at INRA-Versailles [88,89], GABI-KAT [90,91] and SALK [92,93]. The T-DNA insertions are located in 5'-UTR, exon 1 and intron 6, respectively.

### Starch Quantification

Two individual plants (100–400 mg fresh weight) were boiled in 50 ml 80% (v/v) ethanol. The decolored plants were then ground in 80% ethanol with a 2 ml Konte tissue grinder, centrifuged for 10 min at 3,000 g at room temperature. The pellet was washed twice with 80% ethanol. After centrifugation, the insoluble material was suspended in 1 ml of distilled water and boiled for 30 min. Total starch was quantified using a commercial Glc assay kit (catalog no. E0207748; R-Biopharm, Darmstadt, Germany), according to instructions in the kit protocol. The assay employs amyloglucosidase to achieve complete digestion of the starch sample to Glc, and measurement of the Glc by application of a Glc oxidase reagent. The average of six biological replicates, using two individual plants for each replicate, was calculated.

### Software

The computations (normalization, correlation matrix, permutation-based p-values, random graphs generation, hypergeometric distribution) were conducted in R software [94]. The RNA profiles were plotted in MetaOmGraph. The network of metabolic genes in Fig. 2 was visualized using GraphExplore [95]. The pathway data is from MetNetDB database. The R code is available upon request.

## Authors' contributions

WIM designed the study, conducted the bioinformatic analyses and drafted the manuscript. JP carried out the mutant screening and starch staining and quantification. NR implemented the MetaOmGraph software for the analysis. BJN contributed to the interpretation of the data and critical revising and writing of the manuscript. ESW coordinated the study and contributed to its design, interpretation of the results and writing the manuscript. All authors read and approved the final manuscript.

## Supplementary Material

Additional file 1 **Genes used in the study**. Table lists names, Affy IDs, Locus IDs and references for genes used in the study. Genes that form coexpressed modules at 0.6 correlation threshold (Fig. 2B) are depicted on the color background (blue: fatty acid biosynthesis; red: leucine catabolism; green: starch metabolism). Click here for file

Additional file 2 Metadata for the microarray experiments included in the study. Click here for file

Additional file 3 **Genes in the supermodule containing the leucine catabolism pathway**. Table lists genes in the supermodule containing the leucine catabolism pathway (shown in Fig. 8), identified as intersection of lists of all genes that are correlated above 0.5 threshold with each of eight genes from leucine catabolism module. Click here for file

Additional file 4 **Permutation-based support for the correlations in the co-expression network**. Figure shows the permutation-based support for the correlations in the co-expression network of 126 genes in Fig. 2B. For each pair of genes with Pearson correlation above 0.6 this real correlation value (red stars) is compared to the distribution of correlation values obtained in 10,000 permutations of the corresponding expression data vectors (green star, maximum; black star, mean; blue star, minimum; orange bar, values between upper and lower hinges). Click here for file
