# Supplementary material for: Articulation of three core metabolic processes in Arabidopsis: Fatty acid biosynthesis, leucine catabolism and starch metabolism
Source: BMC Plant Biol. 2008 Jul 11;8:76. doi: 10.1186/1471-2229-8-76 (PMC2483283; doi:10.1186/1471-2229-8-76)
Supplement: Additional file 3 — Genes in the supermodule containing the leucine catabolism pathway. Table lists genes in the supermodule containing the leucine catabolism pathway (shown in Fig. 8), identified as intersection of lists of all genes that are correlated above 0.5 threshold with each of eight genes from leucine catabolism module. [file 1471-2229-8-76-S3.doc]

Genes in the supermodule containing the leucine catabolism pathway, shown in Fig. 8, identified as an intersection of lists of all genes that are correlated above 0.5 threshold with each of eight genes from leucine catabolism module.

| **Locus ID** | **Annotation** |
| --- | --- |
| At1g03090 | 3-methylcrotonyl-CoA carboxylase 1 (MCCA) |
| At1g06570 | 4-hydroxyphenylpyruvate dioxygenase (HPD) |
| At1g08630 | L-allo-threonine aldolase-related |
| At1g10070 | branched-chain amino acid aminotransferase 2 / branched-chain amino acid transaminase 2 (BCAT2) |
| At1g15040 | glutamine amidotransferase-related |
| At1g18270 | ketose-bisphosphate aldolase class-II family protein |
| At1g21400 | 2-oxoisovalerate dehydrogenase, putative / 3-methyl-2-oxobutanoate dehydrogenase, putative / branched-chain alpha-keto acid dehydrogenase E1 alpha subunit, putative |
| At1g28260 | expressed protein |
| At1g55510 | 2-oxoisovalerate dehydrogenase, putative / 3-methyl-2-oxobutanoate dehydrogenase, putative / branched-chain alpha-keto acid dehydrogenase E1 beta subunit, putative |
| At1g55810 | uracil phosphoribosyltransferase, putative |
| At1g58180 | carbonic anhydrase family protein |
| At1g76410 | zinc finger (C3HC4-type RING finger) family protein |
| At1g79700 | ovule development protein, putative, similar to ovule development protein AINTEGUMENTA |
| At2g14170 | methylmalonate-semialdehyde dehydrogenase; putative |
| At2g18700 | encodes an enzyme putatively involved in trehalose biosynthesis. |
| At2g40420 | amino acid transporter family protein |
| At2g43400 | encodes a unique electron-transfer flavoprotein:ubiquinone oxidoreductase that is localized to the mitochondrion. |
| At3g06850 | branched chain alpha-keto acid dehydrogenase E2 subunit (din3) |
| At3g13450 | branched-chain alpha-keto acid dehydrogenase E1 beta subunit (DIN4) |
| At3g45300 | isovaleryl-CoA-dehydrogenase (IVD) |
| At3g47340 | asparagine synthetase 1 (glutamine-hydrolyzing) |
| At3g51840 | short-chain acyl-CoA oxidase |
| At4g28040 | nodulin MtN21 family protein |
| At4g30270 | similar to endo xyloglucan transferase |
| At4g34030 | 3-methylcrotonyl-CoA carboxylase 2 (MCCB) |
| At4g35770 | senescence-associated gene |
| At4g38470 | protein kinase family protein |
| At5g16340 | AMP-binding protein, putative |

**Genes in the supermodule containing the leucine catabolism pathway (continued).**

| **Locus ID** | **Annotation** |
| --- | --- |
| At5g20250 | member of glycosyl hydrolase family |
| At5g21170 | 5'-AMP-activated protein kinase beta-2 subunit |
| At5g41080 | glycerophosphoryl diester phosphodiesterase family protein |
| At5g49360 | beta-xylosidase located in the extracellular matrix. |
| At5g49450 | bZIP family transcription factor |
| At5g63620 | oxidoreductase, zinc-binding dehydrogenase family protein, contains PFAM zinc-binding dehydrogenase domain PF00107 |
