# Supplementary material for: Articulation of three core metabolic processes in Arabidopsis: Fatty acid biosynthesis, leucine catabolism and starch metabolism
Source: BMC Plant Biol. 2008 Jul 11;8:76. doi: 10.1186/1471-2229-8-76 (PMC2483283; doi:10.1186/1471-2229-8-76)
Supplement: Additional file 4 — Permutation-based support for the correlations in the co-expression network. Figure shows the permutation-based support for the correlations in the co-expression network of 126 genes in Fig. 2B. For each pair of genes with Pearson correlation above 0.6 this real correlation value (red stars) is compared to the distribution of correlation values obtained in 10,000 permutations of the corresponding expression data vectors (green star, maximum; black star, mean; blue star, minimum; orange bar, values between upper and lower hinges). [file 1471-2229-8-76-S4.doc]

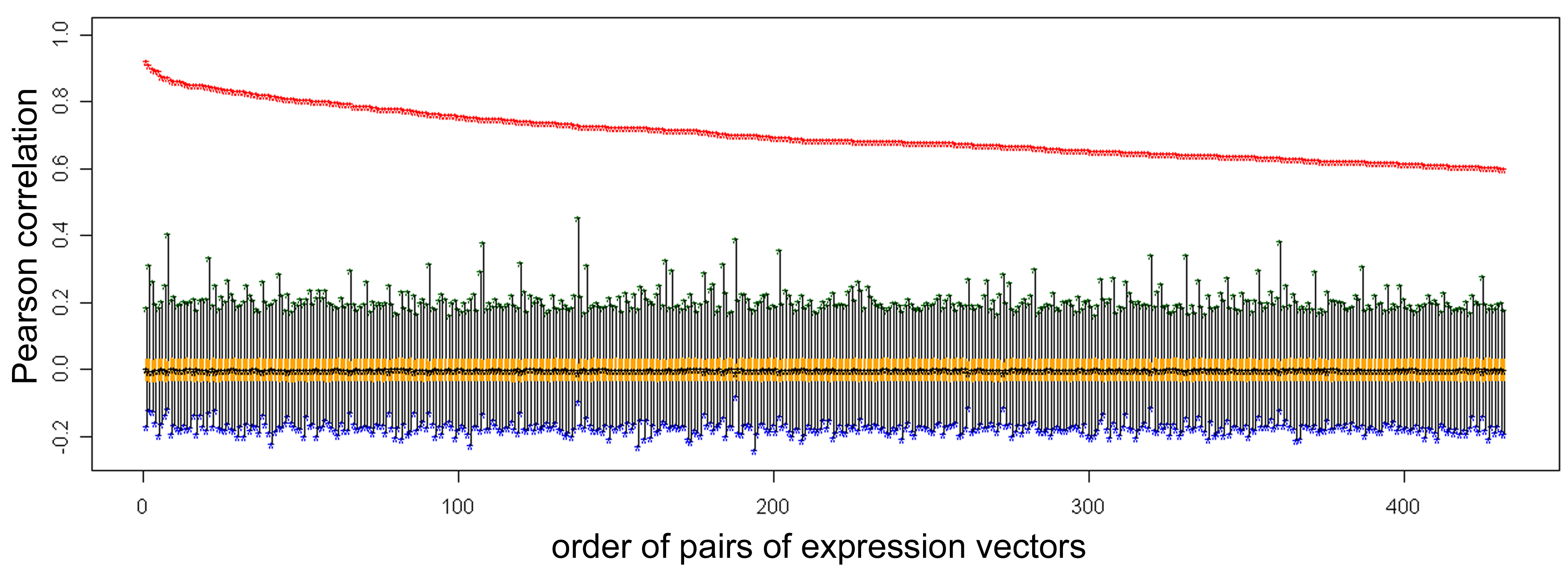


Permutation-based support for the correlations in the coexpression network of 126 genes in Fig. 2B. For each pair of genes with Pearson correlation above 0.6 this real correlation value (red stars) is compared to the distribution of correlation values obtained in 10,000 permutations of the corresponding expression data vectors (green star, maximum; black star, mean; blue star, minimum, orange bar, values between upper and lower hinges).
